# Is self-reporting workplace activity worthwhile? Validity and reliability of occupational sitting and physical activity questionnaire in desk-based workers

**DOI:** 10.1186/s12889-016-3537-4

**Published:** 2016-08-19

**Authors:** Scott J. Pedersen, Cecilia M. Kitic, Marie-Louise Bird, Casey P. Mainsbridge, P. Dean Cooley

**Affiliations:** 1 Active Work Laboratory, Faculty of Education, University of Tasmania, Newnham Dr., Launceston, TAS 7250 Australia; 2 School of Health Sciences, Faculty of Health, University of Tasmania, Hobart, Australia

**Keywords:** Physical activity, Sitting behaviour, Workplace, Exercise

## Abstract

**Background:**

With the advent of workplace health and wellbeing programs designed to address prolonged occupational sitting, tools to measure behaviour change within this environment should derive from empirical evidence. In this study we measured aspects of validity and reliability for the Occupational Sitting and Physical Activity Questionnaire that asks employees to recount the percentage of work time they spend in the seated, standing, and walking postures during a typical workday.

**Methods:**

Three separate cohort samples (*N* = 236) were drawn from a population of government desk-based employees across several departmental agencies. These volunteers were part of a larger state-wide intervention study. Workplace sitting and physical activity behaviour was measured both subjectively against the International Physical Activity Questionnaire, and objectively against ActivPal accelerometers before the intervention began. Criterion validity and concurrent validity for each of the three posture categories were assessed using Spearman’s rank correlation coefficients, and a bias comparison with 95 % limits of agreement. Test-retest reliability of the survey was reported with intraclass correlation coefficients.

**Results:**

Criterion validity for this survey was strong for sitting and standing estimates, but weak for walking. Participants significantly overestimated the amount of walking they did at work. Concurrent validity was moderate for sitting and standing, but low for walking. Test-retest reliability of this survey proved to be questionable for our sample.

**Conclusions:**

Based on our findings we must caution occupational health and safety professionals about the use of employee self-report data to estimate workplace physical activity. While the survey produced accurate measurements for time spent sitting at work it was more difficult for employees to estimate their workplace physical activity.

## Background

Health and wellbeing programs in the workplace are now a common phenomenon in the public health arena and, increasingly, in the subject of research. Typically, evaluations of these programs are achieved through self-report surveys. Some surveys rely on participants’ recall to measure the dependent variable [1, 2]. Recall surveys are popular because they are easy and cost-efficient to administer to large working populations through widespread web-hosting sites. Moreover from a research perspective, recall surveys, if used post hoc the intervention potentially do not influence the dependent variable of interest (i.e., sitting behaviour) [3, 4]. As research inquiry into this area grows there has been an increase in self-report surveys that estimate sitting behaviour [5–10]. This has allowed researchers to describe how much sitting and moving employees do whilst at work. Nonetheless, in contrast to more objective measurements (i.e., accelerometers), there has been criticism regarding the reliability and validity of these surveys [11–16]. The Occupational Sitting and Physical Activity Questionnaire (OSPAQ) [7] is a contemporary addition to the range of surveys used to measure workplace physical activity and sitting time. Researchers have reported high validity coefficients for the OSPAQ [17]. Nonetheless, recent data [18] indicate questionable validity and reliability for the specific measures of the more active behaviours of standing and walking during work. Thus, the purpose of this research was to conduct further assessment of the validity and reliability of the OSPAQ for measuring sitting behaviour and workplace physical activity (standing and walking) across three separate cohorts of desk-based working adults.

## Method

### Study design

The sample was drawn from desk-based employees (*N* = 774) across several Tasmania Government agencies and councils in Tasmania, Australia who voluntarily agreed to participate in an e-health intervention designed to decrease sitting in the workplace. After obtaining ethical approval to conduct this study from the University of Tasmania Social Science Human Research Ethics committee, a random sample of employees were invited to participate in a variety of research studies designed to test the effectiveness of point-of-choice prompts delivered through their personal work computers on a range of health and work behaviour variables. The first three studies (described below) were conducted before the initiation of the voluntary intervention, to test the validity and reliability of the OSPAQ. Participants who responded to an email invitation to participate all provided informed consent through electronic communication in accordance with the University of Tasmania research ethics committee guidelines. All participants were over the age of 18 and employed in either full-time or part-time work at a range of government workplaces across Tasmania. Inclusion criteria required all participants to identify their work as predominantly desk-based and have no prior exposure to sit-stand desk workstations.

### Primary measure

The OSPAQ is a six-item inventory, designed by Chau and colleagues [7], that requires participants to self-report in percentages how much they sit, stand, walk, and perform heavy labour during a typical workday in the last seven days. In addition, participants are asked to provide data (days and hours) regarding how much time they spend at work during a typical work week. Considering the primary focus of this investigation targets desk-based workers, the heavy labour category was omitted from further analysis. Across all three cohorts only two participants indicated a value other than “0” for this category. Both of these responses were given a value of “5 %”, and thus this category and those two participants’ data were excluded from further analysis. This inventory was delivered electronically to all employees in this study through a password protected weblink, which allowed employees to complete the brief survey through their personal work computers during work hours.

### Participants and procedures

#### Cohort 1: criterion validity sample - accelerometry

The first cohort (*n* = 34) was randomly selected from several Department of Health and Human Services workplaces across Tasmania, Australia. The purpose of this component of the study was to test the criterion validity of the OSPAQ using ActivPAL accelerometers to obtain an objective measurement of time spent in the workplace sitting, standing, and walking. Accelerometers are a popular tool to measure criterion validity for sedentary behaviour questionnaires [6]. The ActivPAL (PAL Technologies Ltd, Glasgow, UK) uni-axial accelerometer (15 g, 53 × 35 × 7 mm) was applied by a member of the research team at the midline point of the anterior aspect of the right thigh of each participant to measure distinguishing periods of sitting, standing, and walking. The ActivPal was removed by the same researcher at the end of the working day. Before each data collection, the activity monitors were connected to a personal computer and synchronised using the proprietary software. Postures were inferred from positions of the thigh using proprietary algorithms. Participants’ data were collected over an 8 h workday in their typical working environment. Data collected on the device were then downloaded through a docking station onto the researcher’s personal computer. Through the use of proprietary software time spent sitting, standing, and walking was converted to a percentage of the entire workday.

#### Cohort 2: concurrent validity sample - international physical activity questionnaire

For the second study we recruited a random sample (*n* = 127) of desk-based workers from the Tasmanian Departments of Fire, and Health and Human Services to estimate concurrent validity of the OSPAQ in comparison to an internationally validated [19, 20] measure of sedentary behaviour and physical activity time during work hours. The International Physical Activity Questionnaire (IPAQ) [21] is a tool that measures a range of domains and activities that contribute to a person’s physical activity profile. Amongst them, participants are asked to record the amount of minutes they spend sitting, standing, and walking during a typical workday. Before beginning an e-health intervention participants completed a baseline measure that included the OSPAQ and IPAQ.

#### Cohort 3: reliability sample – test-retest

The final cohort (*n* = 75) was randomly selected from desk-based employees across the Tasmanian Departments of Fire; Health and Human Services; and Primary Industries, Parks, Water and Environment. All of these participants completed the OSPAQ questionnaire one week prior to receiving the e-health intervention. The morning they received the intervention they were asked to complete the OSPAQ before the software program would initiate. The employees were unaware that this second survey was used to measure test-retest reliability of the instrument. All questionnaires were delivered and completed electronically during the employees’ work time. The brevity of this instrument took less than three minutes for the participants to complete, so as not be intrusive to their work responsibilities.

### Data analysis

To illustrate the demographic data from each cohort, participants were asked to report their age, gender, and employment status (Table 1). Full time and part-time employment status was delineated at 38 h of work per week as according to the Australian Government Ombudsman (www.fairwork.gov.au). Criterion validity of the OSPAQ was assessed for each of the three categories (sit, stand, and walk) by comparing the questionnaire responses with the corresponding ActivPal accelerometer values using Spearman’s rho correlation coefficients. The magnitude of these coefficients were interpreted as weak (<0.30), low (0.30–0.49), moderate (0.50–0.69), strong (0.70–0.89) or very strong (≥0.90) [22]. Similarly the concurrent validity of the three OSPAQ categories was measured against the corresponding three IPAQ categories using Spearman’s rho correlation coefficients evaluated against the same magnitude criterion as the criterion validity analysis. Measurement of OSPAQ criterion validity was also determined by comparison to ActivPAL using bias and 95 % limits of agreement (LoA) in accordance with the methods of Bland and Altman [23]. Test-retest reliability of the OSPAQ was assessed by using a two-way mixed model based on absolute agreement to compare intraclass correlation coefficients (ICC) between the two testing times. These coefficients were evaluated as poor (<0.4), good (0.4–0.75), or excellent (>0.75) [24]. To allow for greater generalization, 95 % confidence intervals were provided for all ICC. Data analyses were conducted using IBM SPSS Statistics 21 (SPSS Inc. an IBM Company, Chicago, IL, USA). Bland-Altman analysis was conducted using GraphPad Prism 5 version 5.03 (La Jolla, CA, USA).

**Table 1:** Demographic data for each separate cohort

	Cohort 1	Cohort 2	Cohort 3
Mean age in years (SD)	45.62 (10.96)	44.11 (11.16)	42.87 (11.34)
Gender (Male/Female)	6 / 28	22 / 105	13 / 62
Employment (Full-time/Part-time)	16 / 18	56 / 71	36 /39

## Results

Age, gender, and employment status are presented for each cohort in Table 1.

### Criterion validity

**Fig. 1:**
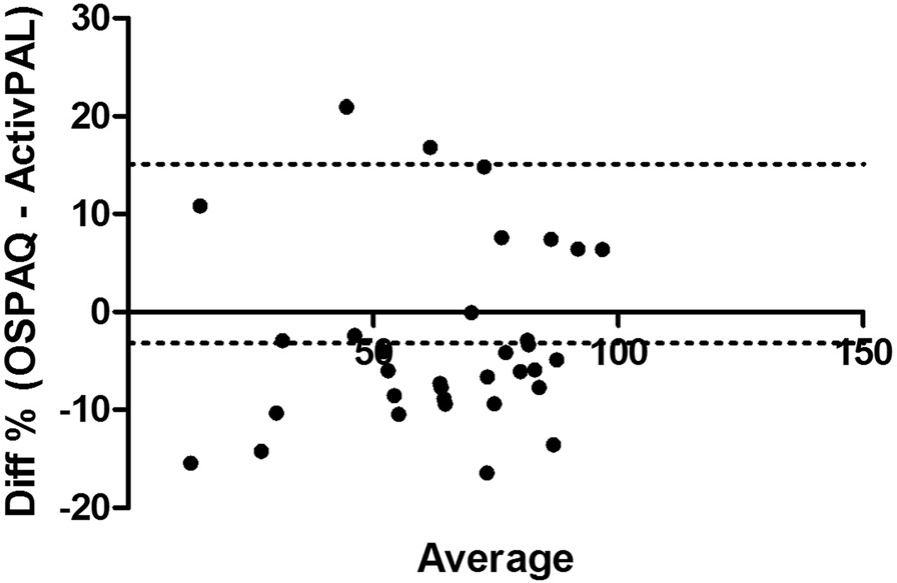
Bland-Altman plot of the difference in reported sitting time (%) between the OSPAQ and ActivPAL. *Dashed lines* represent the mean bias and 95 % LoA

**Fig. 2:**
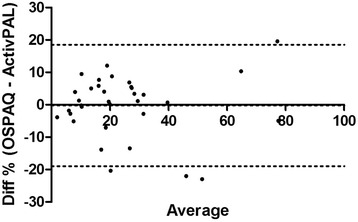
Bland-Altman plot of the difference in reported standing time (%) between the OSPAQ and ActivPAL. *Dashed lines* represent the mean bias and 95 % LoA

**Fig. 3:**
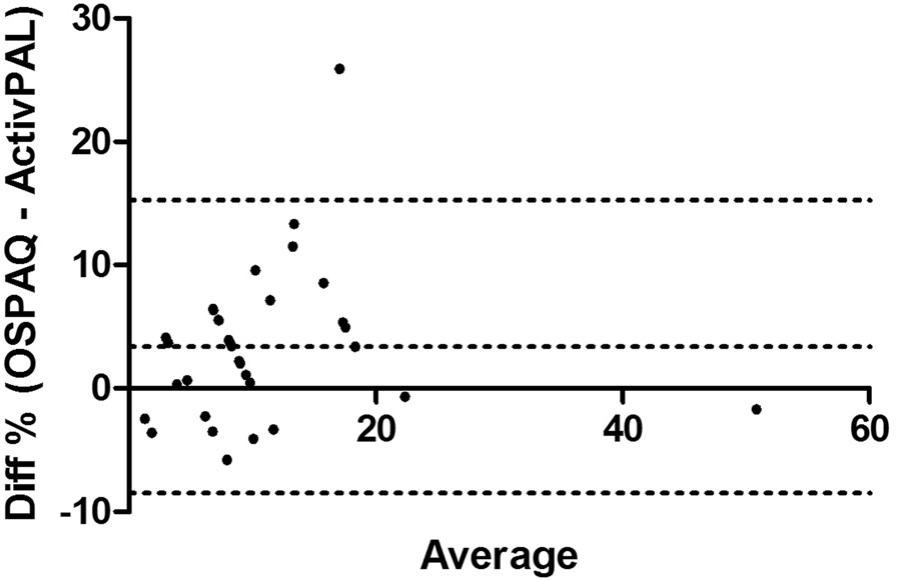
Bland-Altman plot of the difference in reported walking time (%) between the OSPAQ and ActivPAL. *Dashed lines* represent the mean bias and 95 % LoA

Criterion validity for the sitting percentages between the OSPAQ and the ActivPAL was very strong (*rho*_*sit*_ = 0.90). Differences between the two means was nonsignificant, *t*(33) = 1.98, *p* > 0.05. The reported bias was −3.16 % (*SD* 9.32 %) with LoA from −21.4 to 15.1 % (Fig. 1). This criterion validity coefficient for the two standing percentages was strong (*rho*_*sit*_ = 0.84); and also demonstrated nonsignificant differences between the two means, *t*(33) = 0.13, *p* > 0.05. The reported bias was 0.21 % (9.56 %) with LoA from −18.95 to 18.53 % (Fig. 2). Only moderate criterion validity (*rho*_*sit*_ = 0.54) was found between the OSPAQ walking percentage and the ActivPAL walking percentage. The OSPAQ walking percentage (12.44 %) was significantly (*t*[33] = 3.25, *p* = 0.003) greater than the corresponding ActivPAL (9.06 %) percentage. Bland-Altman bias was 3.37 % (6.06 %) with LoA from −8.49 to 15.25 % (Fig. 3). The percentage of work time spent sitting, standing, and walking as quantified by the OSPAQ and ActivPAL are presented in Table 2.

**Table 2:** Descriptive statistics for the OSPAQ and ActivPAL for the determination of criterion validity. Values are mean (SD) percentages of a typical workday

	OSPAQ (%)	ActivPAL (%)
Sitting	62.56 (22.61)	65.72 (21.76)
Standing	25.00 (19.66)	25.21 (19.46)
Walking	12.44 (9.47)	9.06 (8.88)

### Concurrent validity

Concurrent validity between the OSPAQ and the IPAQ was moderate for sitting (*rho*_*sit*_ = 0.62, *p* < 0.0001) and standing (*rho*_*stand*_ = 0.56, *p* < 0.0001), but low for walking (*rho*_*walk*_ = 0.43, *p* < 0.0001). Average bias for sitting was 2.39 % (11.61 %) with LoA from −20.37 to 25.16 %. For standing the bias was −2.87 % (6.66 %) with LoA from −15.94 to 10.19 %. Walking bias was 0.50 % (8.44 %) with LoA-16.05 to 17.05 %. OSPAQ and IPAQ percentages for sitting, standing, and walking time are reported in Table 3.

**Table 3:** Descriptive statistics for the OSPAQ and IPAQ for the determination of concurrent validity. Values are mean (SD) percentages of a typical workday

	OSPAQ (%)	IPAQ (%)
Sitting	79.05 (13.04)	76.65 (12.31)
Standing	9.63 (7.68)	12.5 (8.35)
Walking	11.32 (7.97)	10.83 (6.71)
Hours at Work per Week	34.61 (9.06)	34.75 (8.70)

### Test-retest reliability

Intraclass correlation coefficients for the test-retest reliability of the OSPAQ were low across the three categories (Table 4). The average days at work per week from test 1 and test 2 were 4.68 (0.84) and 4.55 (0.81), respectively. The average time at work per week from test 1 was 35.61 h (7.78 h) and 36.57 h (7.73 h) from test 2.

**Table 4:** Descriptive statistics for the OSPAQ across both tests for the determination of test-retest reliability. Test values are mean (SD) percentages of a typical workday

	Test 1 (%)	Test 2 (%)	ICC (95 % CI)
Sitting	76.85 (14.64)	78.17 (14.09)	0.44 (0.24 – 0.60)
Standing	11.19 (7.89)	10.63 (11.05)	0.37 (0.17 – 0.54)
Walking	11.43 (9.29)	9.91 (5.63)	0.01 (−0.21 – 0.23)

## Discussion

This study aimed to determine the criterion and concurrent validity of the OSPAQ as well as the reliability of this tool in a desk-based population of working adults. The findings from cohort one show that for criterion validity when compared with an ActivPal accelerometer the OSPAQ provided a valid measure of sitting and standing but overestimated the percentage of work time spent walking. Similarly, in cohort two concurrent validity between the OSPAQ and the IPAQ was moderate for sitting and standing, but low for walking. In the third cohort of desk-based workers, reliability of the OSPAQ was poor and as such a more objective measure of physical activity, such as an accelerometer, should be employed for health interventions targeting an increase in workplace physical activity and a reduction in sitting behaviour.

With a very strong and strong relationship between the OSPAQ and ActivPAL for sitting and standing, respectively, the OSPAQ provided a valid measure of static behaviour in a desk-based worker population. Previous correlations of the OSPAQ with ActiGraph accelerometers for occupational sitting and standing time have been weaker than those reported in the present study (*rho* = 0.65 and *rho* = 0.49, respectively) [7]. The accelerometer inclination sensor of the ActivPAL may make this tool more sensitive when compared to the ActiGraph which when positioned on the hip does not appear to distinguish between occupational sitting time and standing time [25]. Even when an ActiGraph was positioned on the thigh, similarly to the present study, Jancey and others reported a Pearson’s correlation coefficient of 0.11 for sitting and 0.61 for standing between the accelerometer and OSPAQ in a population of office-based workers [17]. van Nassau recently reported the LoA between the OSPAQ and ActivPAL prior to the introduction of sit-to-stand workstations, where *rho* ranged from 0.37 to 0.48 for sitting and 0.16-0.20 for standing [25]. Following the introduction of sit-to-stand workstations the agreement between the OSPAQ and ActivPAL was relatively unchanged for sitting (*rho* = 0.35) but increased for standing (*rho* = 0.68), potentially due to an increased awareness of standing behaviour. It is unclear why the agreement between OSPAQ and accelerometry in the present study is stronger than that previously reported, although the workplace environment is likely to be an influencing factor as participants in the study by van Nassau were from a non-government health agency and were not specified as desk-based workers. While the OSPAQ may be a more accessible tool for determining low energy expenditure activity levels in desk-based workers, we suggest that its validity should be verified with accelerometry in the chosen population.

The OSPAQ was not a valid measure of time spent walking when compared to the ActivPAL. The lack of agreement between the OSPAQ and an ActivPal accelerometer for walking is similar to the findings of Chau and others [7] who reported a correlation of 0.29, but less than that reported by Jancey and others (*r* = 0.61) [17]. While Chau did not provide data to determine if the OSPAQ underestimated or overestimated walking, and Jancey showed no systematic bias, participants in the present study tended to overestimate time spent walking. There is an increased awareness to the negative physical [26–29], and mental health [5] implications associated with sitting, especially uninterrupted sitting. This may be one factor that led our participants to overestimate the time spent in incidental physical activity during work hours, and over-report their walking time. Wick and colleagues [18] reported a greater overestimation of walking time (7.7 %) in office workers with the OSPAQ compared to an ActiGraph and this was further exaggerated in workers with a BMI <20 kg/m^2^ using the OSPAQ. It appears that the OSPAQ is suitable to measure time spent sitting, but a more sensitive measure of higher energy expenditure activities, such as an accelerometer, should be employed for interventions designed to monitor walking during the workday.

Mirroring the results of agreement between the OSPAQ and the ActivPAL accelerometer, the OSPAQ and the IPAQ exhibited moderate concurrent validity for sitting and standing, but a low coefficient for walking. Interestingly, previous research has reported an underestimation of sitting time by up to two hours per day using the IPAQ compared to an ActivPAL [30]. While agreement between the OSPAQ and IPAQ in the present study was moderate for low intensity activity, these questionnaires do not assesses the frequency of interruptions to sitting time [10], and as such more sophisticated assessment of posture and physical activity with an accelerometer may be of value.

The reliability of the OSPAQ across all levels of activity was poor, providing further support to use accelerometers as a tool to monitor workplace physical activity. While Chau and colleagues [7] and Jancey and others [17] have reported moderate to very good reliability of the OSPAQ (*ICC* = 0.66–0.90) our findings are similar to those of Wick and colleagues [18] who reported poor reliability of the OSPAQ, particularly for walking (*ICC* = 0.04). With the low ICCs we reported for sitting, standing, and walking the OSPAQ does not appear to be a tool sensitive enough to monitor change in workplace physical activity over time. It would be remiss of us not to mention the issue raised with developing health recommendations based solely on self-report survey data [15]. Self-report data are subject to systematic errors that result in either under- or over-reporting of the dependent variable of interest [16]. We acknowledge that these data were collected from a single population of desk-based workers and as such may not be transferable to other work environments. In conclusion given the results of this study, we would recommend the OSPAQ be modified to address the reliability issues with low forms of physical activity and sitting behaviour. Furthermore, researchers should be cautious in making recommendations for interventions based on data solely garnered from the OSPAQ. We recommend that researchers continue to use reliable and valid survey data in combination with more objective-based data to more accurately describe health behaviour in the workplace.

## Abbreviations

ICC: Intraclass correlation coefficients
IPAQ: International physical activity questionnaire
LoA: Limits of agreement
OSPAQ: Occupational sitting and physical activity questionnaire
